# Factors Associated with GLP-1 Receptor Agonist Use in Patients with Type 2 Diabetes and Established Atherosclerotic Cardiovascular Disease: A Retrospective Propensity-Score Matched Analysis

**DOI:** 10.3390/diseases14020075

**Published:** 2026-02-17

**Authors:** Georgios Vournas, Leonidas Mourgos, Michael Doumas, Evangelos N. Liberopoulos, Kalliopi Kotsa, Theocharis Koufakis

**Affiliations:** 1 Medical School, Aristotle University of Thessaloniki, 54124 Thessaloniki, Greece; g.vournas17@gmail.com; 2 Second Department of Internal Medicine, General Hospital of Athens “G. Gennimatas”, 11527 Athens, Greece; lmourgos@yahoo.gr; 3 Second Propaedeutic Department of Internal Medicine, Medical School, Hippokration General Hospital, Aristotle University of Thessaloniki, 49 Konstantinoupoleos str., 54642 Thessaloniki, Greece; michalisdoumas@yahoo.co.uk; 4 First Department of Propaedeutic Internal Medicine, Medical School, National and Kapodistrian University of Athens, Laiko General Hospital, 11527 Athens, Greece; vaglimp@yahoo.com; 5 Division of Endocrinology and Metabolism and Diabetes Center, First Department of Internal Medicine, Medical School, Aristotle University of Thessaloniki, AHEPA University Hospital, 54636 Thessaloniki, Greece; kalmanthou@yahoo.gr

**Keywords:** type 2 diabetes, cardiovascular disease, GLP-1 receptor agonists, clinical inertia, guidelines

## Abstract

**Background:** Atherosclerotic cardiovascular disease (ASCVD) frequently coexists with type 2 diabetes (T2D), amplifying morbidity and mortality. Glucagon-like peptide-1 receptor agonists (GLP-1RA) confer significant cardiovascular benefits and are recommended for patients with T2D and established ASCVD. However, real-world use may not reflect a complication-driven therapeutic approach. **Methods:** This retrospective study included adults with T2D and established ASCVD (prior myocardial infarction, ischemic stroke, transient ischemic attack, or symptomatic peripheral arterial disease) consecutively admitted to the internal medicine and cardiology departments of a tertiary hospital over a 60-day period. Pre-admission medication use, comorbidities, and laboratory parameters were recorded. Factors associated with GLP-1 RA use were assessed using logistic regression before and after 1:1 propensity score (PS) matching. **Results:** Among 202 eligible patients, 49 (24.3%) were treated with a GLP-1RA. GLP-1RA users were younger (71.9 vs. 77.8 years, *p* < 0.001), had lower hypertension prevalence (61.2% vs. 78.4%, *p* = 0.02), and were more frequently on insulin (69.4% vs. 25.5%, *p* < 0.001) and sodium-glucose cotransporter 2 inhibitors (55.1% vs. 28.1%, *p* = 0.001). After PS matching (48 pairs), demographic and comorbidity differences were attenuated, although insulin remained strongly associated with GLP-1RA therapy (Odds Ratio 11.85, *p* < 0.001). Neither cardiovascular disease burden—captured through the presence of multiple cardiovascular comorbidities—nor renal function were independently associated with GLP-1RA use after adjustment. **Conclusions:** In patients with T2D and established ASCVD, GLP-1RA use was more strongly associated with the intensity of glucose-lowering therapy—particularly insulin use—than with cardiovascular or renal risk profiles. These findings should be interpreted with caution given the retrospective observational design and the limited availability of glycated hemoglobin, anthropometry and diabetes duration data. However, they suggest that, in real-world clinical practice, GLP-1RA prescribing may remain predominantly glucose-centric rather than complication-driven, underscoring the need for improved implementation of contemporary diabetes guidelines.

## 1. Introduction

Atherosclerotic cardiovascular disease (ASCVD) and type 2 diabetes (T2D) frequently coexist and together constitute one of the most challenging combinations in modern clinical practice [1]. Individuals with T2D experience a substantially elevated risk of myocardial infarction, stroke, heart failure, and cardiovascular mortality [2]. This heightened vulnerability is driven by insulin resistance, chronic inflammation, endothelial dysfunction, and accelerated atherosclerotic processes [3]. When ASCVD and T2D occur together, their effects are synergistic rather than additive, resulting in markedly increased morbidity, mortality, and healthcare burden [4]. This convergence underscores the importance of therapeutic strategies that simultaneously address glycemic control and cardiovascular protection.

Over the past decade, glucagon-like peptide-1 receptor agonists (GLP-1RA) have reshaped the management of T2D. Initially introduced as glucose-lowering agents, GLP-1RA demonstrated robust cardiorenal benefits in multiple outcome trials, including reductions in major adverse cardiovascular events and improvements in kidney outcomes [5]. These findings fundamentally shifted the positioning of GLP-1RA from glucose-lowering agents to cardiometabolic therapies. Their dual advantages in improving metabolic outcomes and reducing cardiovascular risk place them at the forefront of contemporary diabetes management, especially for patients with pre-existing ASCVD.

Mechanistically, GLP-1RA improve metabolic control through enhanced glucose-dependent insulin secretion, suppression of glucagon release, delayed gastric emptying, and sustained weight loss, all of which reduce cardiometabolic stress [6]. Importantly, GLP-1RA also exert direct and indirect vascular effects. Experimental and clinical studies have demonstrated improvements in endothelial function, reductions in oxidative stress and systemic inflammation, and favorable modulation of lipid profile [7]. These effects translate into attenuation of atherosclerotic progression and improved arterial compliance. At the myocardial level, GLP-1RA has been associated with improved myocardial glucose uptake, enhanced cardiac efficiency, and protection against ischemia–reperfusion injury [8]. Additionally, GLP-1RA modestly lower systolic blood pressure, likely through natriuresis, improved endothelial nitric oxide availability, and weight reduction [9]. Renal effects, including reductions in albuminuria and preservation of glomerular filtration, further contribute to cardiovascular protection by interrupting the bidirectional relationship between kidney dysfunction and ASCVD [10]. Collectively, these mechanisms provide a biologically plausible explanation for the reductions in major adverse cardiovascular events observed in large outcome trials and support the positioning of GLP-1RA as cardiometabolic agents whose benefits extend well beyond glycemic control.

Reflecting this evolution, recent international guidelines—including those from the American Diabetes Association (ADA) [11], the European Society of Cardiology [12], and the European Association for the Study of Diabetes [13]—recommend the use of GLP-1RA with proven cardiovascular benefit for all patients with T2D and established ASCVD, regardless of baseline glycated hemoglobin (HbA1c) or background therapy. In parallel, sodium–glucose cotransporter-2 inhibitors (SGLT2i) are recommended for those with heart failure or chronic kidney disease (CKD) [11,12,13]. These guideline recommendations represent a shift from a glucose-centric paradigm to a complication-driven, risk-stratified therapeutic approach, emphasizing that physicians should prioritize cardiorenal protection beyond glucose targets.

Despite compelling evidence and strong guideline endorsement, the real-world uptake of GLP-1RA and SGLT2i remains suboptimal. Numerous registry and practice-based studies have demonstrated persistent underuse of these cardioprotective agents among eligible patients, even in those with clear indications such as established ASCVD or CKD [14,15]. Barriers include therapeutic inertia, uncertainties surrounding newer agents, perceived cost or accessibility issues, and clinical habits that continue to prioritize glycemic control over cardiovascular risk reduction. This discrepancy between clinical practice and guideline recommendations underscores the importance of understanding local prescribing patterns and identifying factors that influence treatment decisions. Therefore, the aim of the present study was to evaluate real-world patterns of GLP-1RA use in patients with T2D and established ASCVD, and to identify clinical, demographic, and treatment-related factors associated with GLP-1RA use.

## 2. Methods

### 2.1. Study Design and Setting

This study was conducted as a retrospective observational analysis of adults admitted to the cardiology and internal medicine departments of a large tertiary care hospital in Athens, Greece. The retrospective data collection and chart review were performed during November and December 2025. All parameters were recorded at the time of hospital admission and reflected patients’ pre-admission outpatient status, thereby capturing routine clinical practice rather than in-hospital therapeutic modifications. The study adhered to the principles outlined in the Declaration of Helsinki, and institutional ethical approval for retrospective data collection and analysis was obtained from the responsible ethics committee (approval code: 26; approval date: 11 November 2025). All patient data were anonymized prior to analysis. Because treatment allocation was determined by routine clinical practice, no randomization was performed. The study population was ethnically homogeneous, reflecting the predominantly Caucasian/Greek population served by the institution.

### 2.2. Study Population

Eligible participants were adults with T2D (diagnosed according to the criteria of the ADA [16]) and established ASCVD, defined as a documented history of ischemic heart disease, ischemic stroke, transient ischemic attack (TIA), or symptomatic peripheral arterial disease (PAD). Participants were consecutively included, and the study sample reflected all eligible admissions over a 60-day period, irrespective of the primary reason for hospital admission. Patients were excluded if they had previously been treated with a GLP-1RA but discontinued therapy due to adverse effects, or if they had contraindications to GLP-1RA therapy, including a history of acute or chronic pancreatitis, personal or family history of medullary thyroid carcinoma, multiple endocrine neoplasia type 2, or any other clinician-documented contraindication (e.g., end-stage renal disease or severe gastrointestinal disorders). To ensure the reliability of renal function assessment, patients with acute kidney injury or sepsis at admission were also excluded, as these conditions may cause transient alterations in serum creatinine and estimated glomerular filtration rate (eGFR). Individuals with incomplete documentation of key demographic or clinical variables required for analysis were also excluded.

### 2.3. Data Collection and Recorded Variables

Demographic variables included age and sex. Clinical parameters encompassed the presence or absence of hypertension, dyslipidemia, heart failure, ischemic heart disease, cerebrovascular disease, PAD, and chronic lung disease. Medication use prior to admission was obtained from the national electronic prescribing system, which captures all outpatient prescriptions and reflects patients’ routine pharmacological treatment before hospitalization, rather than medications initiated or modified during the inpatient stay. Medication use prior to admission was recorded for all major classes of glucose-lowering and cardiometabolic therapies. These included insulin, metformin, dipeptidyl peptidase-4 inhibitors, SGLT2i, sulfonylureas, thiazolidinediones, meglitinides, renin–angiotensin–aldosterone system inhibitors (RAASi), and GLP-1RA. During the study period, the GLP-1RA available in Greece included dulaglutide and semaglutide. For the purposes of this analysis, GLP-1RA therapy was evaluated as a class-level exposure (yes/no), and individual agents or dosing regimens were not analyzed separately. Statin therapy was not specifically recorded or analyzed, as lipid-lowering treatment is considered standard of care in patients with established ASCVD and was therefore not expected to meaningfully discriminate GLP-1RA prescribing patterns within this cohort.

Laboratory data collected at admission included serum creatinine, lipid profile, hemoglobin, hematocrit, and electrolytes. eGFR was calculated for all patients using the 2021 CKD-EPI creatinine equation (race-free version), which incorporates age, sex, and serum creatinine [17]. This approach ensured a standardized assessment of renal function across the cohort. Data on HbA1c and body mass index (BMI) were sparsely available and therefore were not included in the primary analyses, reflecting real-world limitations in metabolic documentation at hospital admission. To account for the lack of systematically recorded anthropometric data, the triglyceride-to–HDL cholesterol (TG/HDL) ratio was calculated as a lipid-based surrogate marker of adiposity, given its established association with visceral obesity and insulin resistance [18]. The TG/HDL ratio was explored as an additional covariate in sensitivity analyses evaluating factors associated with GLP-1RA use.

### 2.4. Propensity Score Development and Matching

Given the retrospective and non-randomized nature of the study, propensity score (PS) matching was used to reduce confounding and to approximate the balance of baseline characteristics that would be expected in a randomized comparison. The PS was constructed to estimate each patient’s probability of receiving GLP-1RA therapy based on observed baseline characteristics. The PS model was derived from a multivariable logistic regression that included variables considered potential confounders of treatment selection: age, sex, eGFR, hypertension, dyslipidemia, heart failure, ischemic heart disease, stroke/TIA, PAD, chronic lung disease, and RAASi use. Cardiovascular disease burden was accounted for by including individual cardiovascular comorbidities (ischemic heart disease, cerebrovascular disease, PAD, and heart failure) simultaneously in the PS model, thereby capturing differences between patients with single versus multiple cardiovascular disease manifestations. Notably, other glucose-lowering treatments such as insulin and SGLT2i were intentionally excluded from the PS model because these therapies may lie on the causal pathway between underlying disease severity and clinician decisions regarding GLP-1RA initiation. Including such variables could lead to overadjustment or collider bias in the matched analysis. Patients were matched 1:1 without replacement using a nearest-neighbor approach based on the PS. Covariate balance between the matched groups was assessed using standardized mean differences (SMDs), with values substantially reduced after matching, and all remaining imbalances well below the predefined threshold for large imbalance, as illustrated in Supplementary Figure S1.

### 2.5. Statistical Analysis

Continuous variables were summarized using means and standard deviations, and categorical variables were summarized as frequencies and percentages. Between-group comparisons in the unmatched cohort were performed using independent-samples *t*-tests for continuous variables and χ^2^ or Fisher’s exact tests for categorical variables. SMDs were calculated to evaluate baseline comparability before and after matching. To identify independent factors associated with GLP-1RA therapy, two multivariable logistic regression models were constructed. The first model evaluated the full unmatched cohort, incorporating demographic variables, renal function, comorbidities, and glucose-lowering medications. The second model was applied to the PS-matched cohort and included age, sex, eGFR, cardiovascular comorbidities, and glucose-lowering medications—specifically insulin, metformin, and SGLT2i—to identify variables that remained significantly associated with GLP-1RA use after controlling for confounding. Variables used to construct the PS, including hypertension and dyslipidemia, were not re-entered into the matched regression model to avoid overadjustment and collinearity. Results are presented as odds ratios (OR) with 95% confidence intervals (CI). All analyses were performed using Python (v. 3.11) (pandas and statsmodels libraries).

## 3. Results

The study included 202 patients, of whom 49 (24.3%) were receiving a GLP-1RA. The mean age of the overall cohort was 76.2 ± 9.0 years, and 31.7% were female. GLP-1RA users were significantly younger than non-users, with mean ages of 71.9 and 77.8 years, respectively (*p* < 0.001). The proportion of female patients did not differ between groups (*p* = 0.64). Table 1 presents the characteristics of the study population before and after PS matching.

Renal function also differed modestly between groups. Patients receiving GLP-1RA therapy had a lower mean eGFR (63.9 mL/min/1.73 m^2^) compared with non-users (70.2 mL/min/1.73 m^2^), although this difference did not reach significance (*p* = 0.09). Hypertension was considerably more prevalent among patients not treated with GLP-1RA (78.4% vs. 61.2%), a difference that was significant (*p* = 0.02). Conversely, heart failure was more common among GLP-1RA users (28.6% vs. 15.0%), with a trend toward significance (*p* = 0.06). Other comorbidities, including ischemic heart disease, stroke/TIA, PAD, and chronic lung disease, did not differ meaningfully between groups. Medication patterns demonstrated striking contrasts. Insulin use was significantly more frequent among GLP-1RA users, with 69.4% receiving insulin compared with 25.5% of non-users (*p* < 0.001). SGLT2i use was also more common in the GLP-1RA group (55.1% vs. 28.1%, *p* = 0.001). Metformin use was slightly less common in GLP-1RA users (36.7% vs. 58.2%, *p* = 0.01).

Continuous lipid parameters were available for the entire cohort and are summarized in Table 2. Mean levels of total cholesterol, LDL cholesterol, HDL cholesterol, and triglycerides did not differ significantly between GLP-1RA users and non-users. These findings suggest that differences in lipid-related cardiovascular risk factor levels or control did not account for the observed patterns of GLP-1RA prescribing in this cohort.

In the PS model, younger age (*p* = 0.0006) and lower eGFR (*p* = 0.049) were significantly associated with an increased probability of GLP-1RA use, while hypertension was associated with reduced likelihood of treatment (*p* = 0.002). Dyslipidemia demonstrated a borderline association (*p* = 0.07), whereas other comorbidities were not significant predictors. PS matching produced 48 pairs of GLP-1RA users and non-users, corresponding to 96 matched patients with good covariate balance across demographic and clinical variables. After matching, the mean age of the two groups differed by less than three years (74.0 vs. 71.8 years, *p* = 0.16), and mean eGFR values were nearly identical (*p* = 0.58). Hypertension, dyslipidemia, heart failure, ischemic heart disease, and other comorbidities demonstrated adequate balance after matching (all *p* > 0.20). Despite this improved comparability, marked differences in glucose-lowering therapy persisted after matching. Insulin use remained substantially higher among GLP-1RA users (70.8% vs. 22.9%, *p* < 0.001). The difference in SGLT2i use remained directionally consistent but no longer reached significance in the matched sample (56.3% vs. 45.8%, *p* = 0.27). Improved balance of baseline covariates following PS matching is shown in Supplementary Figure S1.

In the unmatched multivariable logistic regression model (Table 3), several variables were independently associated with GLP-1RA use. Younger age was significantly associated with GLP-1RA treatment (OR 0.94 per year increase in age, *p* = 0.03). Hypertension was inversely associated with GLP-1RA use (OR 0.21, *p* = 0.007). Insulin use was strongly associated with GLP-1RA therapy (OR 5.79, *p* < 0.001). Dyslipidemia demonstrated a borderline association (OR 3.73, *p* = 0.08), while eGFR was not a significant predictor (*p* = 0.25). In the matched logistic regression model, insulin use remained the strongest independent predictor of GLP-1RA therapy, with an OR of 11.85 (*p* < 0.001). Neither age (*p* = 0.44), sex (*p* = 0.66), nor eGFR (*p* = 0.16) was associated with GLP-1RA use. Individual cardiovascular comorbidities—including heart failure (*p* = 0.32) and ischemic heart disease (*p* = 0.37)—were also not independently associated with GLP-1RA therapy. Accordingly, patients with multiple coexisting cardiovascular conditions were not more likely to receive GLP-1RA treatment than those with a single cardiovascular manifestation. SGLT2i use demonstrated a positive but nonsignificant association (*p* = 0.29).

The TG/HDL ratio did not differ significantly between GLP-1RA users and non-users (*p* = 0.34) and was not independently associated with GLP-1RA use in multivariable analysis (OR 1.00, 95% CI 0.98–1.02; *p* = 0.82). These findings suggest that, within the limits of available lipid-based surrogate markers, differences in adiposity or insulin resistance are unlikely to explain the observed GLP-1RA prescribing patterns. Overall, the results indicate that although several demographic and clinical factors are associated with GLP-1RA use in the unmatched cohort, the only robust independent predictor after accounting for confounding through PS matching is insulin therapy, reflecting substantial differences in treatment intensity between patients receiving GLP-1RA and those not treated with this medication class.

**Figure 1:**
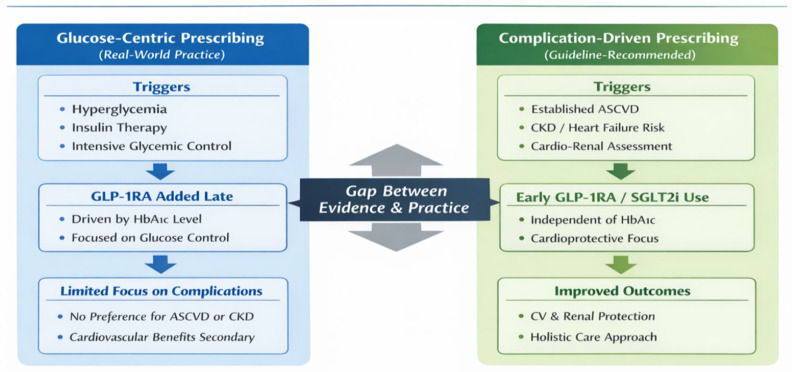
Comparison of glucose-centric versus complication-driven prescribing in patients with type 2 diabetes and established ASCVD. Abbreviations: ASCVD, atherosclerotic cardiovascular disease; BMI, body mass index; CKD, chronic kidney disease; CV, cardiovascular; GLP-1RA, glucagon-like peptide-1 receptor agonist; HbA1c, glycated hemoglobin; HbA1c, glycated hemoglobin; SGLT2i, sodium–glucose cotransporter-2 inhibitor.

Beyond patient-level clinical characteristics, GLP-1RA use in this cohort should be interpreted within the broader context of the healthcare system and prescribing environment. During the study period, access to GLP-1RA therapy was influenced by national reimbursement policies, prescribing regulations, and physician discretion, which may have contributed to conservative use of newer agents. These system-level factors, together with clinician prescribing habits and therapeutic inertia, likely shaped real-world treatment patterns and may have limited risk-based adoption of GLP-1RA therapy, independent of individual patient cardiovascular or renal profiles. Figure 1 summarizes the key findings of the present study, highlighting the lack of preferential GLP-1RA use among patients with greater cardiovascular or renal risk and the predominance of glycemia-driven treatment decisions in real-world clinical practice.

## 4. Discussion

In this retrospective analysis of 202 adults with T2D and established ASCVD, several important findings emerged. Despite strong guideline recommendations supporting the use of GLP-1RA in this high-risk population, overall uptake was low, with only 24.3% of eligible patients receiving GLP-1RA therapy. GLP-1RA use was primarily associated with markers of glycemic treatment intensification, most notably concurrent insulin therapy, which remained the strongest determinant of treatment allocation both before and after rigorous PS matching. In contrast, after adjustment for age, renal function, and comorbidities, neither specific cardiovascular disorders nor differences in overall cardiovascular disease burden—reflected by the coexistence of multiple cardiovascular comorbidities—were independently associated with GLP-1RA use, and renal function likewise showed no residual association. Given the single-center, inpatient-based design, these findings primarily reflect local real-world prescribing practices within a tertiary care setting and should be interpreted as context-specific rather than universally generalizable.

The novel contribution of this study lies in providing country-specific real-world evidence from Greece on GLP-1RA use among patients with T2D and established ASCVD, a population for whom these agents are strongly recommended by contemporary guidelines. In the absence of robust regional data, our findings offer insight into current prescribing patterns within the Greek healthcare setting by integrating patient-level clinical characteristics, medication use, and PS-adjusted analyses. By contextualizing GLP-1RA utilization in routine practice, this study contributes nationally relevant evidence that can inform future efforts to align clinical decision-making with evolving cardiometabolic care paradigms.

Although GLP-1RA have demonstrated robust cardioprotective effects independent of glycemic control [19], the demographic and clinical characteristics of GLP-1RA users in this study do not suggest preferential allocation to patients with more advanced cardiovascular disease. Renal function, despite being an important consideration in diabetes management, did not independently influence treatment patterns after adjustment, even though recent evidence from the FLOW trial [20] demonstrated a significant reduction in kidney outcomes and cardiovascular death with semaglutide, underscoring a persistent gap between emerging renal outcome data and real-world prescribing behavior. These patterns echo previous reports demonstrating a divergence between updated guideline recommendations and routine clinical practice, even among patients at high cardiovascular risk [21]. The absence of differences in continuous lipid parameters between GLP-1RA users and non-users further supports the interpretation that GLP-1RA prescribing in this real-world cohort was not guided by objective measures of cardiovascular risk factor levels or control. Notably, LDL cholesterol levels remained above guideline-recommended targets in both groups, suggesting that lipid risk factor control was suboptimal across the cohort. Together, these findings indicate that treatment allocation appeared more closely aligned with glycemic treatment escalation rather than comprehensive cardiovascular risk optimization, consistent with a predominantly glucose-centric approach to diabetes management and a broader pattern of clinical inertia in high-risk patients.

Instead, the strongest driver of GLP-1RA use—both before and after PS matching—was concurrent insulin therapy. In the matched cohort, insulin users had nearly 12-fold higher odds of receiving a GLP-1RA, even after careful adjustment for demographic characteristics, renal function, and cardiovascular comorbidity profiles. This striking and persistent association suggests that GLP-1RA initiation commonly occurs in the context of advanced diabetes requiring treatment escalation, often alongside multidrug regimens [22]. The continued clustering of SGLT2i therapy among GLP-1RA users, although no longer significant after matching, further supports the presence of a broader treatment intensification pattern rather than isolated therapeutic decisions. It should be emphasized, however, that insulin treatment represents an imperfect surrogate for diabetes severity, as it may reflect treatment intensification strategies, clinician preference, or healthcare system factors rather than glycemic burden alone.

Taken together, these findings suggest that, at least in this clinical setting, the transition from a glucose-centric paradigm toward a complication-driven model of diabetes management has not yet been fully realized [23]. Despite strong evidence and guideline recommendations supporting GLP-1RA use for cardiovascular risk reduction, prescribing patterns appear to be shaped primarily by glycemic burden and the perceived need for additional glucose-lowering therapy rather than by cardiovascular or renal risk stratification. This potential misalignment between evidence and practice highlights important opportunities for targeted clinician education and system-level interventions aimed at promoting earlier and more systematic use of GLP-1RA in patients most likely to benefit from their cardiometabolic effects [24].

The evolving concept of the cardio–renal–metabolic syndrome underscores the interconnected nature of ASCVD, CKD, heart failure, obesity, and T2D, and highlights the need for therapeutic strategies that address this continuum holistically rather than in isolation [25]. GLP-1RA and SGLT2i uniquely fulfill this role, as both drug classes exert complementary and synergistic benefits across multiple organ systems. While SGLT2i primarily target hemodynamic and renal pathways—reducing intraglomerular pressure, improving heart failure outcomes, and slowing renal disease progression—GLP-1RA predominantly modulate metabolic, inflammatory, and atherosclerotic processes [26]. Together, they represent a paradigm shift toward integrated cardiometabolic risk reduction.

In this context, the combined use of GLP-1RA and SGLT2i should be viewed not merely as escalation of glucose-lowering therapy, but as a comprehensive disease-modifying strategy. However, the findings of the present study are consistent with real-world prescribing patterns that may not yet fully align with this holistic framework. Instead, GLP-1RA and SGLT2i use appears to remain closely tied to glycemic considerations rather than being systematically deployed to mitigate cardiometabolic risk. Closing this gap will require a conceptual shift in clinical practice—from treating isolated disease components to addressing the shared pathophysiology of cardio–renal–metabolic syndrome using therapies designed to modify its full spectrum [27].

The association of younger age with greater GLP-1RA use in the unmatched cohort may reflect perceptions regarding treatment suitability, as clinicians may be more inclined to prescribe injectable agents or weight-focused therapies to younger patients [28]. Conversely, concerns about polypharmacy, frailty, sarcopenia, cost, or tolerability may cause hesitancy in prescribing GLP-1RA in older individuals, even when cardiovascular risk is greater [29]. The lower prevalence of hypertension among GLP-1RA users may reflect more than simple clinical differences between groups. Hypertension often serves as a marker of long-standing cardiometabolic disease, multimorbidity, and treatment complexity. Clinicians may be less inclined to initiate newer injectable therapies in patients who appear frailer, more comorbid, or already burdened by extensive polypharmacy [30]. In addition, GLP-1RA are frequently perceived as better suited to younger or more metabolically driven phenotypes, whereas hypertensive patients tend to represent a more vascular phenotype [31]. These factors, combined with therapeutic inertia and misalignment between cardiovascular risk and prescribing behavior, may help explain why patients with hypertension were less likely to receive GLP-1RA therapy despite being among those most likely to benefit. The lack of observed sex-based differences in GLP-1RA prescribing suggests that, within this high-risk population, treatment decisions were not influenced by gender, although further studies in more diverse cohorts are warranted.

Beyond the variable-specific interpretations discussed above, the unmatched cohort analyses should be viewed primarily as descriptive and contextual. These unadjusted findings capture real-world prescribing patterns and illustrate the baseline differences between patients receiving and not receiving GLP-1RA that motivated the use of PS matching. The attenuation of several associations after matching highlights the role of confounding by indication in observational data and reinforces the importance of the matched analyses for identifying independent determinants of GLP-1RA use. Accordingly, while the unmatched results provide clinically informative insight into clinician behavior and treatment selection in routine practice, the PS-matched cohort represents the appropriate basis for inference regarding factors independently associated with GLP-1RA prescribing.

### Strengths and Limitations

The present study has several strengths. It incorporates detailed clinical, comorbidity, and laboratory data from a well-defined population of individuals with established ASCVD. The application of PS matching improved comparability between treatment groups, enhancing the validity of the observed associations. The inclusion of both unmatched and matched regression models provides a robust examination of factors independently associated with GLP-1RA use.

However, several limitations should be acknowledged. The retrospective design introduces the potential for unmeasured confounding and limits causal inference. Important clinical factors, such as duration of diabetes, BMI, waist circumference, glycemic trajectory, and patient preferences, were not available and may have influenced treatment decisions. Obesity was sparsely recorded and likely underrepresented. This limitation reflects real-world clinical practice, where anthropometric parameters are frequently under documented in routine medical records, even among high-risk cardiometabolic patients [32]. To partially address this gap, we explored the TG/HDL ratio as a lipid-based surrogate marker of adiposity and insulin resistance. The TG/HDL ratio did not differ between GLP-1RA users and non-users and was not independently associated with GLP-1RA use, a finding that further supports the interpretation that GLP-1RA prescribing in this cohort was driven primarily by glycemic treatment intensity rather than by differences in adiposity or insulin resistance. Nonetheless, the absence of systematically collected anthropometric data limits granularity and underscores the need for improved documentation of obesity-related parameters to support a more holistic, complication-driven approach to diabetes management in routine care.

Missing HbA1c values were also common in our cohort, reflecting real-world clinical practice patterns. This observation is aligned with recent evidence demonstrating that HbA1c is frequently not measured at hospital admission in patients with diabetes, as highlighted by a recent European multicenter study [33], and underscores the need for improved documentation, systematic metabolic assessment, and overall quality of diabetes care in the inpatient setting. Nevertheless, the high prevalence of insulin use among GLP-1RA recipients may serve as a surrogate marker of more advanced diabetes progression and greater disease severity, partially compensating for the lack of systematic HbA1c data in the interpretation of treatment patterns. However, insulin use represents a pragmatic indicator of treatment intensity rather than a definitive measure of diabetes severity and should therefore be interpreted with caution. Although PS matching substantially improved covariate balance, the reduced size of the matched cohort inevitably limited statistical power and may have constrained the detection of more modest associations. This trade-off between precision and internal validity is inherent to matching-based approaches and should be considered when interpreting the results. Nevertheless, the use of SMDs and balance diagnostics, rather than reliance on *p*-values alone, supports the robustness of the matched analyses. The single-center nature of the study and its focus on hospitalized patients may limit the generalizability of the findings to other healthcare systems, outpatient settings, or regions with different organizational structures and reimbursement policies. Nevertheless, the observed prescribing patterns are consistent with reports from other real-world studies, suggesting that similar implementation gaps may exist across diverse clinical contexts. Despite these limitations, the study provides meaningful insight into real-world GLP-1RA prescribing behaviors and raises important considerations regarding the alignment of clinical practice with evidence-based guidelines.

## 5. Conclusions

In this real-world cohort, GLP-1RA prescribing appeared to be driven primarily by glycemic treatment intensity rather than by cardiovascular or renal risk considerations, despite strong evidence supporting their cardioprotective benefits. Interpretation of these findings should take into account the limited availability of key metabolic and clinical variables, including HbA1c, BMI, and duration of diabetes, which reflects real-world documentation practices but may constrain risk stratification. Greater efforts are needed to promote a complication-driven, holistic cardio–renal–metabolic treatment strategy and to improve the integration of cardioprotective therapies into everyday clinical decision-making for high-risk patients.

## Figures and Tables

**Table 1:** Characteristics of participants before and after propensity score matching.

Variable	No GLP-1RA (*n* = 153)	GLP-1RA (*n* = 49)	*p*-Value	SMD	Matched No GLP-1RA (*n* = 48)	Matched GLP-1RA (*n* = 48)	SMD
Age (years)	77.8 ± 8.1	71.9 ± 10.0	<0.001	0.65	74.0 ± 9.3	71.8 ± 10.0	0.24
Female (%)	32.7%	29.2%	0.64	0.07	22.9%	29.2%	0.19
eGFR (mL/min/1.73m^2^)	70.2 ± 23.9	63.9 ± 31.2	0.09	0.23	61.6 ± 22.4	64.4 ± 31.7	0.09
Hypertension (%)	78.4%	61.2%	0.02	0.38	58.3%	60.4%	0.04
Dyslipidemia (%)	71.2%	87.8%	0.02	0.38	75.0%	75.0%	0.00
Heart failure (%)	15.0%	28.6%	0.06	0.33	31.3%	27.1%	0.09
Ischemic heart disease (%)	44.4%	49.0%	0.57	0.06	45.8%	47.9%	0.04
Stroke/TIA (%)	31.4%	34.7%	0.67	0.07	29.2%	36.5%	0.08
Peripheral arterial disease (%)	15.0%	14.3%	0.92	0.02	14.6%	14.3%	0.01
Chronic lung disease (%)	14.4%	8.2%	0.25	0.20	6.3%	8.3%	0.00
Insulin use (%)	25.5%	69.4%	<0.001	1.08	22.9%	70.8%	1.06
SGLT2i use (%)	28.1%	55.1%	0.001	0.58	45.8%	56.3%	0.22
Metformin use (%)	58.2%	36.7%	0.01	0.45	54.2%	37.5%	0.35

Continuous variables are presented as mean ± standard deviation, and categorical variables are presented as number (percentage). *p*-values refer to comparisons between GLP-1RA users and non-users in the unmatched cohort. SMD values > 0.25 indicate large imbalance before propensity score matching. Abbreviations: GLP-1RA: glucagon-like peptide-1 receptor agonist; SMD: standardized mean difference; eGFR: estimated glomerular filtration rate; TIA: transient ischemic attack; SGLT2i: sodium–glucose cotransporter-2 inhibitor.

**Table 2:** Lipid parameters in GLP-1RA users and non-users.

Parameter	GLP-1RA Users	Non-Users	*p*-Value
Total cholesterol (mg/dL)	123.2 ± 37.0	125.0 ± 40.7	0.814
LDL cholesterol (mg/dL)	68.5 ± 33.5	69.2 ± 35.8	0.927
HDL cholesterol (mg/dL)	30.1 ± 7.5	32.8 ± 12.5	0.150
Triglycerides (mg/dL)	135.7 ± 111.8	122.9 ± 82.7	0.536

Values are presented as mean ± standard deviation. Abbreviations: GLP-1RA: glucagon-like peptide-1 receptor agonist.

**Table 3:** Multivariable logistic regression analyses for factors associated with GLP-1RA use.

Variable	Unmatched Model OR (95% CI)	*p*-Value	Matched Model OR (95% CI)	*p*-Value
Age (per year)	0.94 (0.89–0.99)	0.03	1.02 (0.96–1.09)	0.44
Female sex	0.95 (0.37–2.44)	0.91	1.29 (0.42–3.99)	0.66
eGFR (per mL/min/1.73 m^2^)	0.99 (0.97–1.01)	0.25	1.01 (0.99–1.04)	0.16
Hypertension	0.21 (0.07–0.65)	0.007	—	—
Dyslipidemia	3.73 (0.86–16.20)	0.08	—	—
Heart failure	1.44 (0.49–4.25)	0.51	0.53 (0.15–1.84)	0.32
Ischemic heart disease	1.08 (0.43–2.69)	0.87	1.75 (0.51–5.98)	0.37
RAASi use	0.63 (0.24–1.67)	0.35	0.63 (0.23–1.75)	0.38
Insulin use	5.79 (2.24–14.96)	<0.001	11.85 (3.79–37.10)	<0.001
Metformin use	0.74 (0.30–1.82)	0.51	1.02 (0.34–3.13)	0.97
SGLT2i use	2.08 (0.86–5.07)	0.11	1.72 (0.62–4.75)	0.29

Abbreviations: GLP-1RA: glucagon-like peptide-1 receptor agonist; OR: odds ratio; CI: confidence interval; eGFR: estimated glomerular filtration rate; RAASi: renin–angiotensin–aldosterone system inhibitor; SGLT2i: sodium–glucose cotransporter-2 inhibitor.

## Data Availability

The data presented in the study are available on request from the corresponding author. The data are not publicly available due to privacy restrictions of the Greek National Health System.

## Supplementary Materials

The following supporting information can be downloaded at https://www.mdpi.com/article/10.3390/diseases14020075/s1, Figure S1: Covariate balance before and after propensity score matching.
