# Supplementary material for: Factors Associated with GLP-1 Receptor Agonist Use in Patients with Type 2 Diabetes and Established Atherosclerotic Cardiovascular Disease: A Retrospective Propensity-Score Matched Analysis
Source: Diseases. 2026 Feb 17;14(2):75. doi: 10.3390/diseases14020075 (PMC12939290; doi:10.3390/diseases14020075)

Supplementary Figure S1. Covariate balance before and after propensity score matching. Standardized mean differences (SMDs) for baseline covariates before (open circles) and after (filled triangles) propensity score (PS) matching. Vertical dashed line indicates perfect balance (SMD = 0). After matching, covariate balance improved across included variables, supporting the comparability of GLP-1RA users and non-users in the matched cohort.

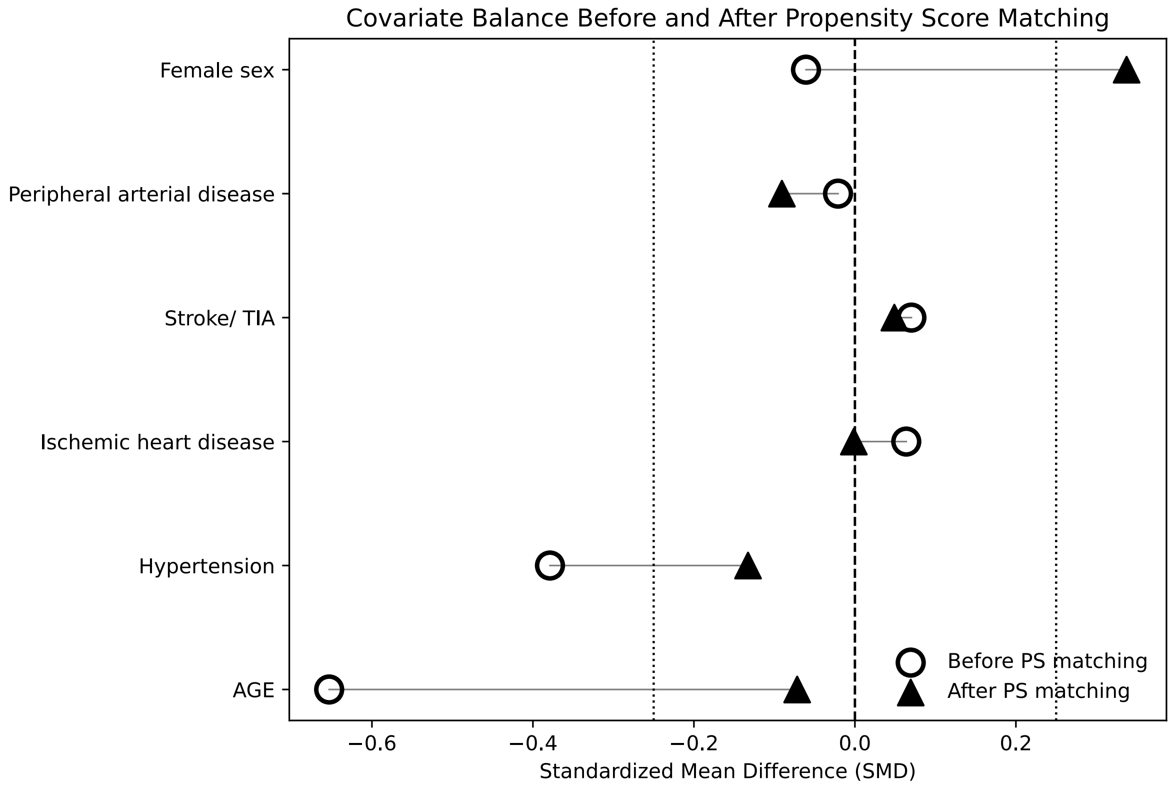

Supplement: Supplementary file 1 [file diseases-14-00075-s001.zip › diseases-4080970-supplementary.pdf]
